# Virtual Reality and Three‐Dimensional Printing of Craniopharyngioma: A Case Report Proposing an Innovative Model of Pediatric Endocrine Care to Enhance Patient Engagement

**DOI:** 10.1002/ccr3.70948

**Published:** 2025-09-23

**Authors:** Valeria Calcaterra, Maurizio Vertemati, Francesco Rizzetto, Laura G. Valentini, Andrea Saladino, Cassandra Gazzola, Sara Zanelli, Francesca Colombo, Arianna Barbotti, Francesco Cavaliere, Tommaso Santaniello, Paolo Milani, Gianvincenzo Zuccotti

**Affiliations:** ^1^ Department of Internal Medicine and Therapeutics University of Pavia Pavia Italy; ^2^ Pediatric Department Buzzi Children's Hospital Milano Italy; ^3^ Department of Biomedical and Clinical Science University of Milano Milano Italy; ^4^ Centro Interdisciplinare Materiali e Interfacce Nanostrutturati (CIMaINa) Università Degli Studi di Milano Milano Italy; ^5^ Dipartimento di Radiologia ASST Grande Ospedale Metropolitano Niguarda Milano Italy; ^6^ Postgraduate School of Diagnostic and Interventional Radiology Università Degli Studi di Milano Milano Italy; ^7^ Department of Neurosurgery Fondazione IRCCS Istituto Neurologico Carlo Besta Milan Italy; ^8^ Dipartimento di Fisica “A. Pontremoli” Università Degli Studi di Milano Milano Italy

**Keywords:** 3D printing, craniopharyngioma, head mounted display, pediatric endocrinology, virtual reality

## Abstract

Craniopharyngiomas, while histologically benign, are well‐known for their difficult clinical course and the potential for substantial morbidity. In craniopharyngioma, head‐mounted display‐based immersive virtual reality and 3D printing can offer an innovative approach to enhancing the quality of multidisciplinary endocrine care, promoting patient engagement and participation in their treatment.

## 1 Introduction

Craniopharyngiomas are rare, histologically benign brain tumors that develop in the pituitary–hypothalamic area and can occur in both children and adults [1, 2, 3, 4]. The histological type differs between childhood‐onset cases (where the vast majority of tumors are of the adamantinomatous type) and adult‐onset cases, which are either of the papillary type, almost exclusively found in adults, or adamantinomatous [5, 6]. Typically, craniopharyngiomas consist of a combination of solid mass and fluid‐filled cysts. As they grow, they can exert pressure on adjacent brain structures such as the pituitary gland, optic chiasm, optic nerves, and cerebral fluid spaces [1, 4].

Consequently, craniopharyngiomas can impair growth and various brain functions, including cognition and vision. In both children and adults, common presenting symptoms include headaches, visual disturbances, nausea, and vomiting. Growth failure is frequently observed in children, while adults often present with hypogonadal symptoms. Increases in thirst or polyuria and weight gain can also manifest [1, 4]. These signs or symptoms caused by the tumor may begin before diagnosis and continue for months or years. Primary amenorrhea rarely presents as a symptom at the onset [5, 7].

Treatment for craniopharyngioma is not specific and mostly noncurative, and frequently includes surgery, which may achieve gross total or partial resection, followed by radiation therapy [1, 2, 3, 5]. While some approaches, such as posterior hypothalamus‐sparing surgery, seem to yield better outcomes in terms of postsurgical complications, the optimal surgical strategy remains debatable [8, 9]. There remains apprehension regarding the potential for aggressive surgical approaches to lead to elevated rates of endocrinologic, metabolic, and behavioral complications, depending on the exact location of the tumor and its size [10].

Virtual reality (VR) technologies using head‐mounted display (HMD) and three‐dimensional (3D) printing of organ models have been recently introduced for completion of preoperative planning in adults [11]. This approach faces limitations in pediatric settings, primarily due to the challenges of integrating patient‐specific imaging data to create anatomically precise and detailed VR models, a process that requires substantial technical and medical expertise. The pediatric age range includes infants to adolescents; it is essential to have dedicated models, as those built for adults would not account for the potential differences related to developmental processes.

Herein, we present an innovative approach to pediatric endocrine care using HMD‐based immersive VR and 3D printing in a case of craniopharyngioma treated with an endoscopic endonasal approach (EEA). The model is used in fostering patientengagement.

## 2 Case History/Examination

A 15‐year‐old girl was admitted to our Pediatric Unit with a 2‐week history of headaches originating from the left eye, with a fluctuating pattern and variable intensity, accompanied by decreased vision and a restricted visual field with right medial hemianopia. Primary amenorrhea was also reported.

The patient leads a healthy lifestyle, with no exposure to tobacco, alcohol, or illicit drugs. She follows a regular diet and has no known dietary restrictions. Physical activity is within normal limits for age. There is no history of chronic illnesses, surgeries, or hospitalizations. Family history is negative for neurological, endocrine, or genetic disorders.

At evaluation, the girl's weight and height were 48.8 kg (10°–25° percentile) and 152 cm (3°–10° percentile), respectively, with a BMI of 21.1 kg/m^2^ (50° percentile); puberty stage according to Tanner was 4.

Cardiothoracic and abdominal clinical examination was unremarkable.

Ophthalmological, neuropsychiatric, endocrine, and imaging assessments were performed.

## 3 Conclusion and Results

During the ophthalmologic examination with optical coherence tomography (OCT), a slight reduction in ganglion cells and nerve fiber layer was observed in the right eye. In the left eye, there was asymmetry in the ganglion cells and a reduction in nerve fiber thickness. Visual acuity tests revealed 4/10 vision in both the left and right eyes, with visual field loss measured at 91.6% in the right eye and 85.8% in the left eye. No abnormal changes in intraocular pressure were observed.

**FIGURE 1:**
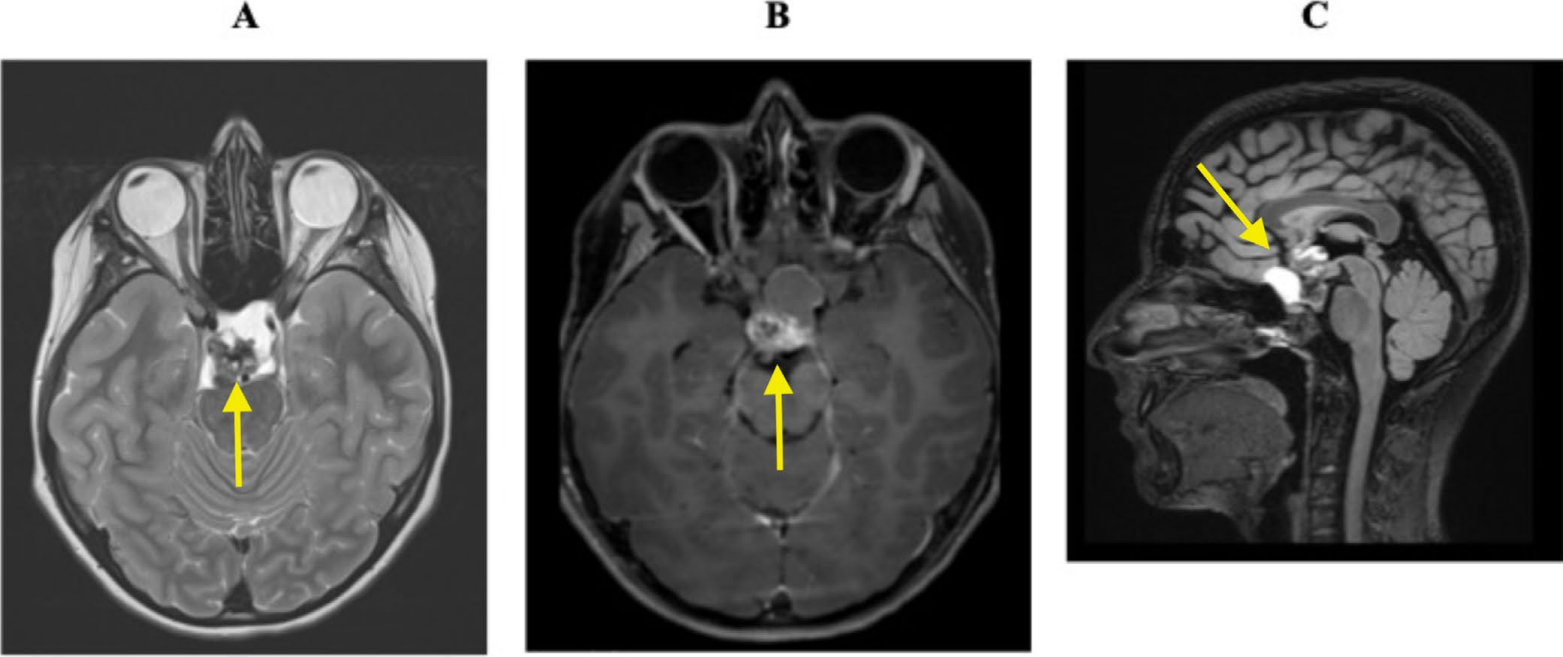
Cerebral MRI depicting the patient's craniopharyngioma. Panel A: The T2‐weighted image (A) shows a suprasellar expansive lesion with a complex internal structure containing both cystic and solid components (yellow arrow). Panel B and C: In the post‐contrast T1‐weighted image (B), the tumor solid components demonstrates pronounced enhancement (yellow arrow). Notably, the lesion displaces the optic chiasm and exerts mass effect on the third ventricle, hypothalamic area, and pituitary stalk, as illustrated in the sagittal FLAIR image (C, yellow arrow). These findings correlate with the neurological and endocrine dysfunctions observed in the patient.

At radiologic examinations, the presumptive diagnosis of craniopharyngioma measuring 35 × 30 × 30 mm was established (Figure 1). Radiological hypothalamic damage was assessed using the Müller grading system, which classified the lesion as Grade II [12]. A critical compromise of the hypothalamic area and the optic chiasm was identified, along with compression of normal *pituitary* tissue, despite a normal hormonal pattern for gonadotropin and other pituitary hormones (Table 1).

**TABLE 1:** Clinical and hormonal features at the diagnosis and post‐surgery.

	At diagnosis	Post‐surgery 1 month	Post‐surgery 6 months
Weight (kg)	48.8 (10°–25° p.le)	47.1 (10°–25° p.le)	79.2 (97° p.le)
Height (cm)	152 (3°–10° p.le)	152.5 (3°–10° p.le)	154 (10° p.le)
Body mass index (kg/m^2^)	21.1 (50° p.le)	20.3 (25°–50° p.le)	33.4 (97° p.le)
Cortisol (48–195 μg/L)	259	192	< 1
ACTH (7.2–63.3 ng/L)	12.9	< 1.5	< 1.5
ADH (< 14 ng/L)	nd	< 0.57	nd
IGF‐1 (140–480 μg/L)	162	61	148
TSH (0.47–3.41 mU/L)	2.3	0.04	< 0.01
FT3 (3.6–5.7 pmol/L)	3.7	2.9	5.7
FT4 (10–14.3 pmol/L)	12	10.5	13.9
17β‐Estradiol (31–533 ng/L)	29	< 5	< 5
FSH (1.7–134 U/L)	7.7	< 0.3	< 0.3
LH (1–95.6 U/L)	4.4	< 0.3	< 0.3
Prolactin (5–23 μg/L)	16	17	Not detected
Sodium level (135–145 mmol/L)	142	141	140
Potassium level (3.5–5 mmol/L)	4.5	3.7	4.2

Abbreviations: ACTH, adrenocorticotropic hormone; ADH, antidiurec hormone; FSH, Follicle‐stimulating hormone; FT3, free triiodothyronine; FT4, free thyroxine; IGF, insulin growth factor; LH, luteinizing hormone.

A highly detailed 3D digital model of the patient's lesion was obtained using routine contrast‐enhanced MRI images obtained from a 3 T scanner (MAGNETOM Vida, Siemens Healthineers, Germany) before surgery. 3D time‐of‐flight (ToF) angiography and contrast‐enhanced 3D T1 sequences were used to segment the vessels, while 3D T1‐ and T2‐weighted sequences, including FLAIR (Fluid‐Attenuated Inversion Recovery), were used to segment the other structures of interest. All sequences used had a thin slice thickness (≤ 1 mm).

**FIGURE 2:**
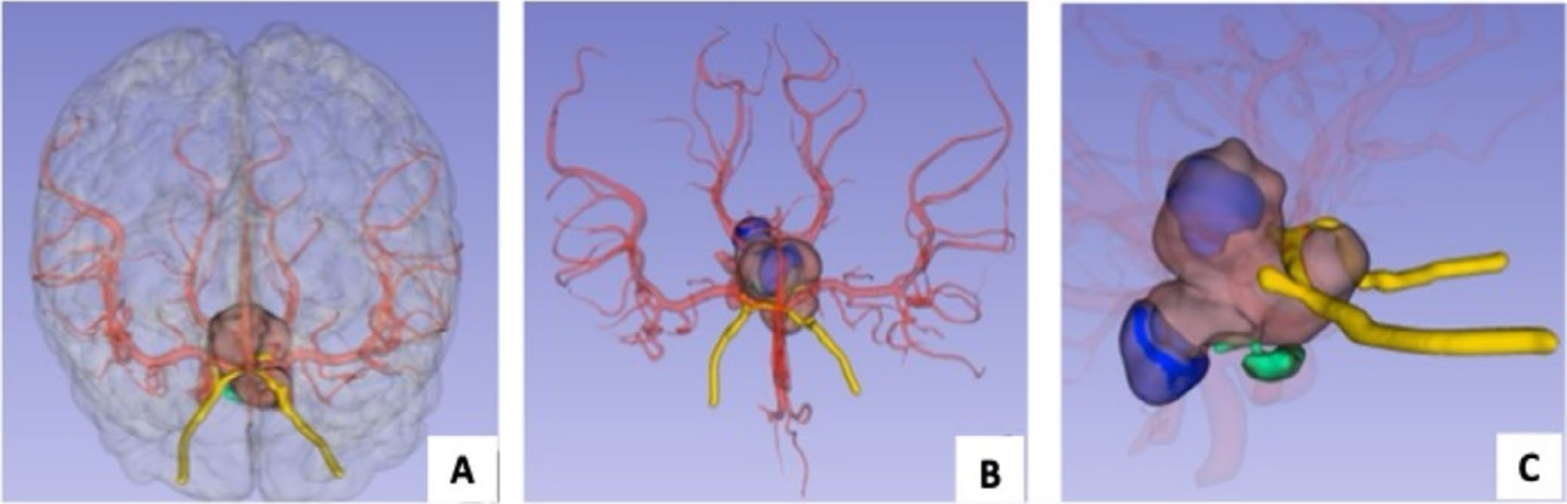
Panel A: Frontal view of the surface model of craniopharyngioma with nervous and vascular structures obtained from segmentation of medical imaging. Panel B: Frontal view of the surface model of craniopharyngioma with nervous and vascular structures obtained from segmentation of medical imaging. Brain has been removed. Panel C: Surface model of craniopharyngioma with nervous and vascular structures obtained from segmentation of medical imaging (yellow: optic nerve; green: hypophysis; brown: solid mass; blue: fluid‐filled cysts).

The MRI images were retrieved from the institutional PACS (Picture Archiving and Communication System), anonymized, and saved in DICOM format. The exported files were then loaded into 3D Slicer v.5.2.1 (www.slicer.org), a free and open‐source software package for image analysis and advanced visualization. Automatic and semi‐automatic tools pre‐built in the software were used to segment the relevant anatomical structures, performing manual corrections when necessary under the supervision of an experienced radiologist. These segmentations were used to obtain 3D surface models. The segmentations were then exported into Stereolithography (.stl) files and, using an in‐house developed plugin for 3D Slicer, were loaded into the Oculus Quest v.1 (META Inc., Menlo Park, CA, USA), an all‐in‐one HMD equipped with an OLED display with a 1440–1600 pixel per eye resolution and a refresh rate of 72 Hz was utilized (Figure 2, Panel A–C; Figure 4 Panel A, C).

**FIGURE 3:**
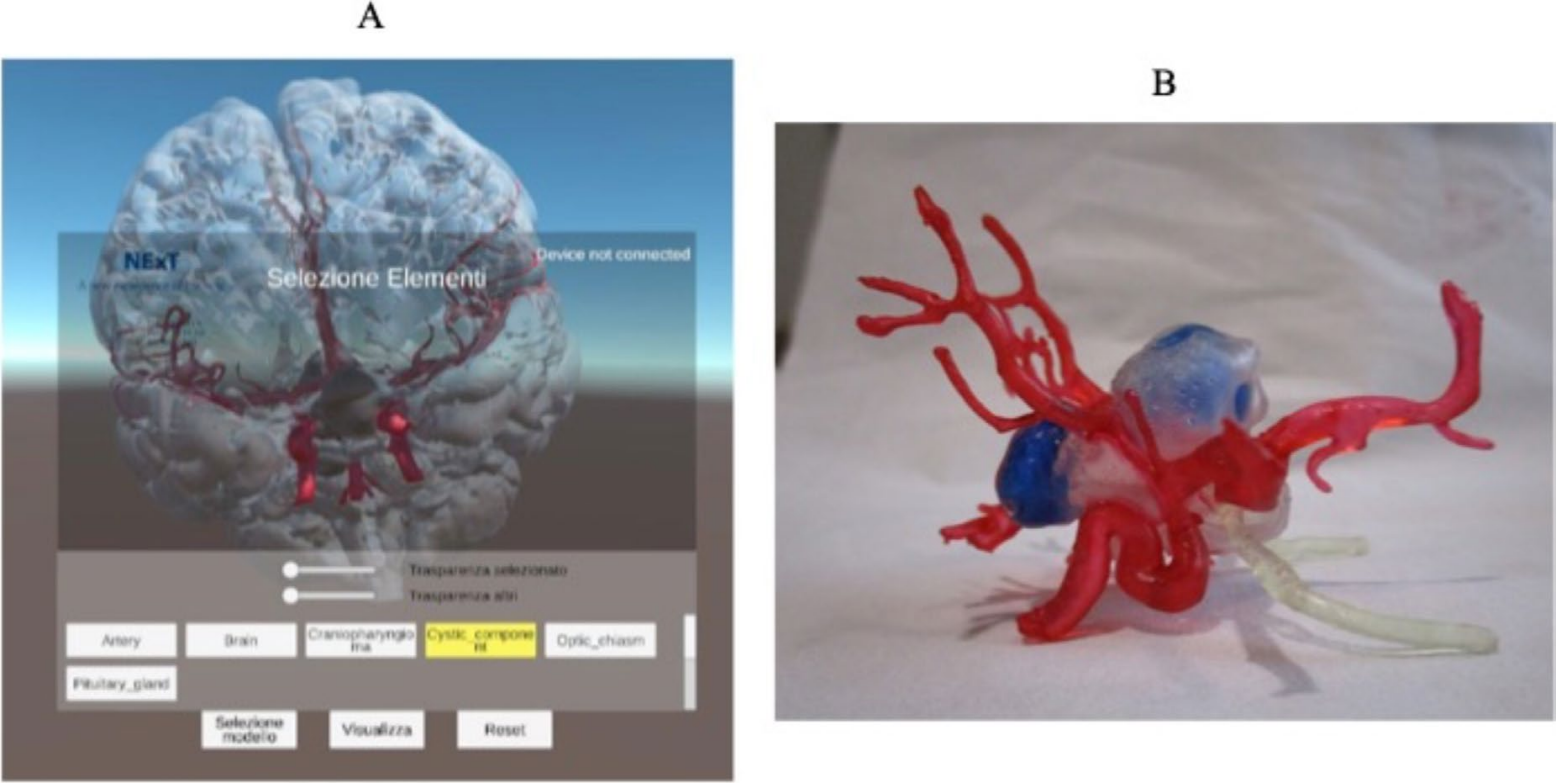
Panel A. Screenshot of the 3‐dimensional scene shown to the user in the HDMI Virtual Reality Environment with the menu interface. Panel B. The craniopharyngioma 3D‐printed model. The virtual models were exported in STL files for 3D printing.

A previously developed application [13, 14] was employed to create a dedicated VR environment for visualizing the VR scene and interacting with the 3D models. The application allowed for motion, rotation, zooming, and adjusting transparency levels of the 3D VR models through a wireless controller (Figure 3).

The same digital models were used to obtain a 3D printed model through additive manufacturing, thereby allowing a multidisciplinary approach, neurosurgical planning, and simulation. In brief, we replicated the life‐sized craniopharyngioma by adapting an additive manufacturing approach developed for a previous work [15] (Figure 3).

In this case, we relied on a laser‐based VAT photo‐polymerization technique, employing suitable transparent and flexible resins to fabricate the various anatomical structures (tumor, vascular structures, and hypophysis) in a single monolithic part, focusing on minimizing building supports by optimizing part orientation during printing. The entire manufacturing process, including post‐processing and curing, took approximately 7 h. The central lesion was designed with two internal cavities filled with blue dye to reproduce the embedded cysts, emphasizing their position relative to surrounding structures. Additionally, various dyes were used to color vessels, optical nerves, and hypophysis, enhancing contrast between contiguous structures to for easier anatomical inspections by physicians and surgeons. The final 3D printed model was assembled by combining the craniopharyngiomas and the surrounding structures, in particular the optic nerves and hypophysis.

The virtual model was used to facilitate discussions with both the parents and the patient. It provided a more effective illustration of the details of the lesion, making it easier to explain complex concepts such as the surrounding anatomy and its relationships with other structures. As a result, the patient was able to ask focused and specific questions about the potential risks and complications associated with the resection. Additionally, various questions will be posed regarding hypothalamo‐pituitary dysfunction and central obesity.

Following a comprehensive discussion and consensus among all involved treatment disciplines, the operation was conducted. The girl was operated on through EEA, and gross total resection was achieved.

## 4 Outcome and Follow‐Up

**FIGURE 4:**
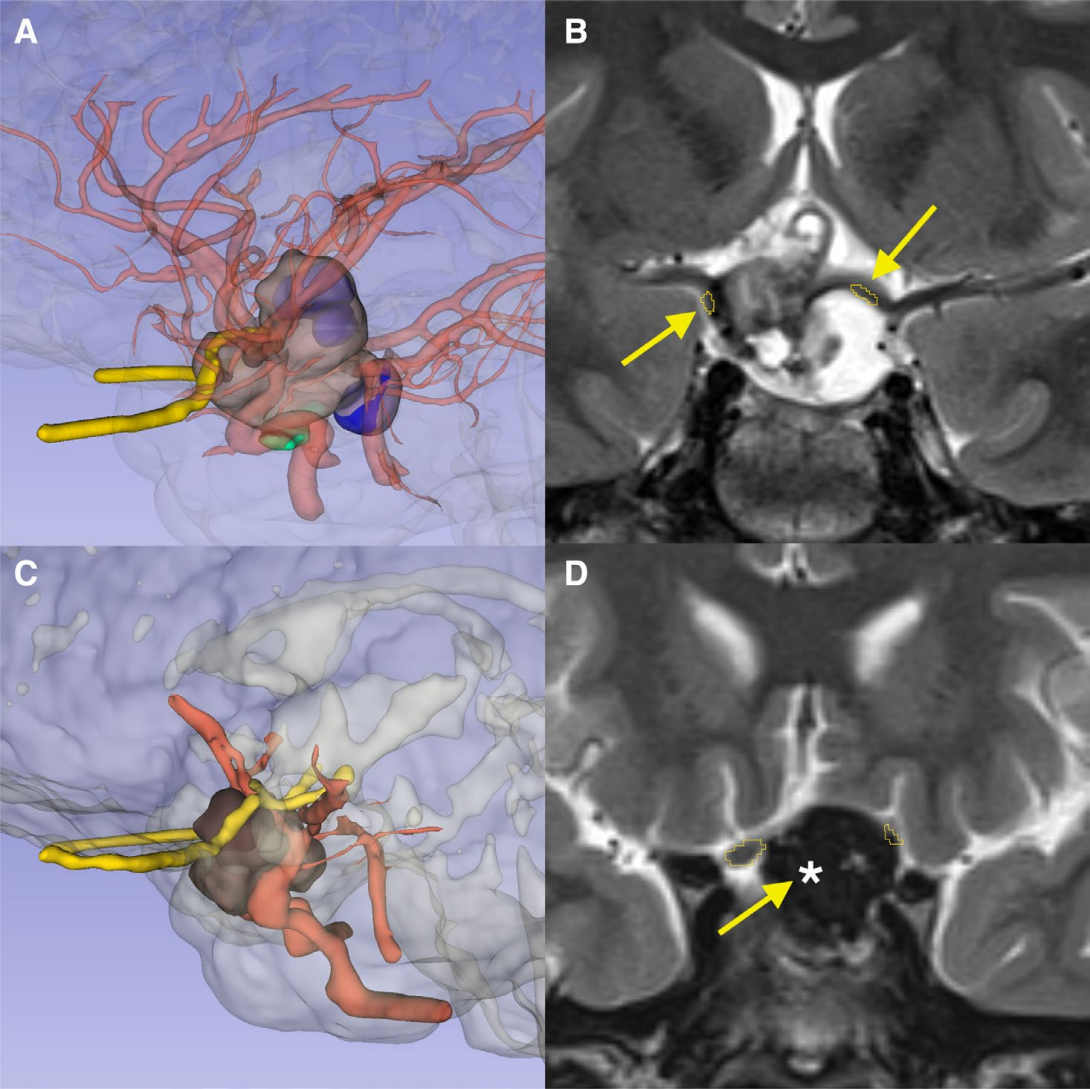
Preoperative coronal fat‐saturated T2‐weighted image (A) and corresponding 3D reconstruction (B) shows the craniopharyngioma compressing and displacing the hypothalamus, optic nerves (FIGURE arrow and outlined in yellow), and surrounding critical structures. Postoperative MRI (C) and 3D reconstruction (D) demonstrate near‐complete restoration of anatomy, with no detectable tumor remnants. The surgical cavity (yellow arrow and asterisk) remains in close proximity to the optic nerves and hypothalamus.

The resection of the lesion was complete, as confirmed by postoperative imaging (Figure 4, Panel A–D). The resulting surgical cavity inevitably affected the hypothalamic region, highlighting the delicate balance required to achieve radical tumor removal while minimizing damage to this critical structure. No immediate postoperative complications developed.

There was an improvement in both visual acuity (6/10 in the right eye and 10/10 in the left eye) and visual field (43% in the right eye and 46.6% in the left eye). Multiple endocrinological deficits occurred and were treated in the postoperative period (corticosteroid, vasopressin, L‐thyroxine). As shown in Table 1, a rapid weight gain associated with hypothalamic obesity was noted. No memory or cognitive dysfunction was recorded.

Nutritional and exercise counseling was provided. Long‐term monitoring and lifelong hormone replacement therapy (estro‐progestin, growth hormone) have been planned. An optimal level of patient therapeutic adherence was observed, contributing to improved treatment outcomes and overall management of the condition.

In Table 1, clinical and hormonal data before and after surgical treatment are presented.

## 5 Differential Diagnosis

The histological characteristics of adamantinomatous craniopharyngioma were determined. Other tumors occurring in the sellar and parasellar regions (pituitary adenomas, germinoma, and low‐grade glioma) were excluded.

## 6 Discussion

We presented a case of craniopharyngioma in a girl with visual symptoms and primary amenorrhea, utilizing a HMD‐based immersive VR environment and 3D printing for surgical planning. These technologies enhance the value of 3D digital reconstructions of medical imaging for improved visualization and interactive navigation of the intricate pituitary–hypothalamic anatomy, bolstering surgeon confidence and skills to answer to the traps in the operating room and improving patient's empowerment.

Craniopharyngioma is a rare brain tumor predominantly found in children, though it can also impact adults. Craniopharyngiomas remain a significant challenge to manage. Despite being classified as histologically benign neoplasms (WHO grade I), they present with a variety of symptoms at diagnosis and are associated with considerable morbidity and mortality due to their aggressive behavior. These tumors frequently infiltrate surrounding brain structures, including the hypothalamus, pituitary gland, and optic nerves, and they may also obstruct the third ventricle. Additionally, treatment‐related damage to hypothalamo‐pituitary structures and functions contributes to complications [2, 16].

Gross total resection is associated with the best recurrence‐free survival outcomes; however, it is crucial to avoid irreversible hypothalamic damage and hypothalamo‐pituitary dysfunction. Early postoperative and long‐term endocrine management is therefore mandatory [16].

As observed in our patient, significant weight gain frequently occurs within the first 12 months following surgery. Various studies have highlighted risk factors for obesity following surgery, such as hypothalamic damage, high BMI at diagnosis, hydrocephalus, surgical techniques, and radiotherapy. Nevertheless, only hypothalamic injury has been consistently confirmed as a major contributing factor [17]. The risk of severe obesity rises in proportion to the extent of hypothalamic damage, particularly when the medial and posterior nuclei are involved, as these areas play a key role in regulating melanocortin pathways. Damage to these regions often leads to hyperphagia, central resistance to insulin and leptin, diminished sympathetic activity, lower energy expenditure, and greater fat accumulation in adipose tissue. These combined effects drive rapid weight gain, with hyperphagia causing persistent feelings of hunger.

Several grading systems have been proposed to predict the likelihood of postoperative obesity, utilizing MRI evaluations to assess preoperative and postoperative hypothalamic involvement [12, 18, 19, 20, 21, 22, 23, 24]. However, MRI grading systems lack objective criteria for quantifying the extent of hypothalamic damage, making them potentially subjective and influenced by the specifics of the surgical procedure [25].

To date, most treatment approaches for hypothalamic obesity have shown limited success, with long‐term outcomes remaining poor. Nevertheless, emerging pharmacological therapies offer a promising avenue for future intervention.

Visual impairment is one of the most common presentations of craniopharyngioma, occurring in 62%–84% of cases at the time of diagnosis [26]. Although postoperative visual outcomes and recovery are generally favorable, complete recovery may not always occur, as observed in our patient. Postoperative visual outcomes are influenced by a complex interplay of factors, including preoperative deficits, tumor size and location, patient age, duration of symptoms, and the surgical techniques employed [26, 27].

Over the past two decades, the utilization of 3D virtual models has significantly expanded as a valuable tool for physicians, with a majority of research focusing on evaluating their role in surgical contexts [28, 29, 30, 31].

These technologies provide a tangible, three‐dimensional representation of the patient's topographic anatomy, offering beneficial adjunctive imaging tools that add value to the preoperative planning and navigation process [29, 30, 31]. Additionally, this approach can be useful for surgical training simulation.

In the era of digital transformation, pediatricians may find particular interest in integrating VR and innovative care models also into their daily clinical pediatric practice, especially in diseases requiring multidisciplinary clinical management, such as craniopharyngioma.

In patients with craniopharyngioma, pediatric endocrinologists and neurosurgeons collaborate closely, and the involvement of the patient is crucial in optimizing the therapeutic pathway. Their cooperation and understanding are necessary for achieving successful treatment outcomes. This innovative approach offers a significant opportunity to deepen the pediatric endocrinologist's comprehension of the patient's lesion, involve patients and their family members in surgical care, and elucidate the specific risks associated with craniopharyngioma surgery, such as the potential loss of hormonal production by the pituitary gland or hypothalamus and anticipate postoperative therapeutic requirements.

Clinical studies on the advantages of these new visualization techniques and 3D printing in pediatrics remain relatively limited, focusing on the use of these approaches in a surgical context [2, 28, 29, 30, 31]. However, both medical staff and patients stand to gain from the opportunities presented by 3D presentations also in an endocrinological pediatric setting.

As showed in our case, patient education is important during the informed consent process. The aid of interactive 3D reconstruction proves beneficial in fostering patient engagement and participation in their care, thereby facilitating acceptance of therapies. In this individual case, we did not specifically evaluate the extent to which 3D imaging improved patient compliance, understanding, or risk assessment using dedicated questionnaires or other instruments. However, the use of 3D imaging was intended to enhance visualization and communication, which may have indirectly contributed to these outcomes. Additionally, the utilization of 3D models enhances patients' understanding and comprehension of medical information, reducing fear and anxiety, and thereby improving their satisfaction with the care received [32].

In Table 2, the advantages of using HMD‐based VR models and 3D printing of organ models in neurosurgery for craniopharyngioma were summarized.

**TABLE 2:** Advantages of using virtual reality technologies using head‐mounted display and three‐dimensional printing of organ models in neurosurgery for craniopharyngioma.

**For surgeon**
Topographic anatomy highlights Preoperative planning and navigation Intraoperative navigation Training simulation
**For patient**
Improvement in understanding and comprehension of medical information Patient education (informed consent process) Patient engagement

In conclusion, craniopharyngiomas, while histologically benign, are well‐known for their difficult clinical course and the potential for substantial morbidity. In craniopharyngioma, HMD‐based VR models and 3D printing may offer an innovative approach to improve the quality of multidisciplinary endocrine care and the patient empowerment. 3D printed patient‐specific anatomical models have proven useful in enhancing understanding of complex surgical procedures, their associated risks, benefits, and alternatives. Exploring the value of virtual 3D models across various disciplines, considering also the role of this approach in engaging the patient in their healing process, could be beneficial from an outcomes perspective.

## Author Contributions

**Valeria Calcaterra:** conceptualization, investigation, methodology, writing – original draft, writing – review and editing. **Maurizio Vertemati:** conceptualization, formal analysis, investigation, methodology, supervision, writing – original draft, writing – review and editing. **Francesco Rizzetto:** formal analysis, investigation, methodology, writing – original draft. **Laura G. Valentini:** investigation, writing – original draft, writing – review and editing. **Andrea Saladino:** investigation, writing – original draft. **Cassandra Gazzola:** investigation, methodology, writing – original draft. **Sara Zanelli:** investigation, methodology, writing – original draft. **Francesca Colombo:** investigation, methodology, writing – original draft. **Arianna Barbotti:** investigation, methodology, writing – original draft. **Francesco Cavaliere:** investigation, methodology, writing – original draft. **Tommaso Santaniello:** investigation, methodology, writing – original draft. **Paolo Milani:** formal analysis, funding acquisition, investigation, methodology, writing – original draft, writing – review and editing. **Gianvincenzo Zuccotti:** conceptualization, funding acquisition, methodology, supervision, writing – original draft, writing – review and editing.

## Ethics Statement

All procedures performed in this study were in accordance with ethical standards of the Helsinki declaration.

## Consent

Written informed consent was obtained from the patient and parents to publish this report in accordance with the journal's patient consent policy.

## Conflicts of Interest

The authors declare no conflicts of interest.

## Data Availability

The data that support the findings of this study are available from the corresponding author upon reasonable request.
